# The SARS-CoV-2 B.1.351 lineage (VOC β) is outgrowing the B.1.1.7 lineage (VOC α) in some French regions in April 2021

**DOI:** 10.2807/1560-7917.ES.2021.26.23.2100447

**Published:** 2021-06-10

**Authors:** Bénédicte Roquebert, Sabine Trombert-Paolantoni, Stéphanie Haim-Boukobza, Emmanuel Lecorche, Laura Verdurme, Vincent Foulongne, Mircea T. Sofonea, Samuel Alizon

**Affiliations:** 1 Laboratoire Cerba, Saint Ouen L’Aumône, France; 2 Laboratoire de Virologie, CHU de Montpellier, Montpellier, France; 3 MIVEGEC, CNRS, IRD, Université de Montpellier, Montpellier, France; 4 These authors contributed equally to this article.

**Keywords:** COVID-19, virus, variant, RT-PCR, epidemiology, statistical modelling, immune evasion

## Abstract

To assess SARS-CoV-2 variants spread, we analysed 36,590 variant-specific reverse-transcription-PCR tests performed on samples from 12 April–7 May 2021 in France. In this period, contrarily to January–March 2021, variants of concern (VOC) β (B.1.351 lineage) and/or γ (P.1 lineage) had a significant transmission advantage over VOC α (B.1.1.7 lineage) in Île-de-France (15.8%; 95% confidence interval (CI): 15.5–16.2) and Hauts-de-France (17.3%; 95% CI: 15.9–18.7) regions. This is consistent with VOC β’s immune evasion abilities and high proportions of prior-SARS-CoV-2-infected persons in these regions.

‘Variants of concern’ (VOC) are severe acute respiratory syndrome coronavirus 2 (SARS-CoV-2) phenotypically distinct lineages that are associated with major epidemiological or clinical shifts. To date, four have been classified as such by the World Health Organization (WHO) [1]. The first, VOC α, which corresponds to Pango lineage B.1.1.7, nextstrain clade 20I/501Y.V1, and GISAID clade/lineage GRY, is currently causing the majority of infections in Europe and North America [2], whereas, the second, VOC β (Pango lineage: B.1.351; nextstrain clade: 20H/501Y.V2; GISAID clade/lineage: GH/501Y.V2) is the most common variant in South Africa [3]. The third variant, VOC γ (Pango lineage P.1; nextstrain clade: 20J/501Y.V3; GISAID clade/lineage: GR/501Y.V3) dominates in Brazil and South America [4] and the fourth VOC δ (Pango lineage: B.1.617.2; nextstrain clade: 21A/S:478K; GISAID clade/lineage: G/452R.V3) caused a major epidemic wave in India [5]. The outcome of the (indirect) competition between variants is yet open. In France, the early introduction of VOC β in some regions makes it particularly important to monitor the spread of different variants [6].

## PCR testing of SARS-CoV-2-positive clinical samples for variants

Since January 2021, the national guideline is to test all clinical samples that are positive for SARS-CoV-2 with an additional reverse-transcription (RT)-PCR to detect mutations indicative of certain variants [7,8]. Since April 2021, this variant-specific RT-PCR targets the N501Y mutation, which is shared by VOCs B.1.1.7, B.1.351 and P.1, and the E484K mutation, which is found in VOCs B.1.351 and P.1, as well as the variant of interest (VOI) B.1.525 (WHO: η; nextstrain clade: 20A/S484K; GISAID clade/lineage: G/484K.V3) [1], but not in B.1.1.7.

We used the ID SARS-CoV-2/N501Y/E484K Quadruplex assay (ID Solution, Grabels, France) to test 53,687 SARS-CoV-2 positive samples collected between 12 April and 7 May 2021 in 13 French regions, with the majority of samples coming from the Île-de-France region (Table 1). Some of the total samples (7–8%, Table 1) originated from hospitals (mostly hospitalised patients), the rest from the general population. We only analysed data from individuals aged from 5 to 80 years to minimise in the analysis the weight of preschool children and elderly persons in long-term care facilities. 17.3% of the tests could not be interpreted; this was mainly because the cycle threshold (Ct) value was too high to ensure an equal sensitivity for the N501Y and E484K targets. To avoid biasing the variant screening, all tests with Ct values strictly above 30, including those where a lineage could be assigned, were ignored (31.8%; 17,097/53,687). Overall, we analysed 68.2% (36,590/53,687) of all the samples tested (Table 1).

**Table 1:** Characteristics of the samples analysed via variant-specific reverse-transcription PCR, France, 12 April–7 May 2021 (n = 36,590)

Characteristics of the samples		Quantitative data
Age in years of the persons providing a sample; number of samples (95%CI)		38 (10–73)
Origin of the samples	General population; number of samples (%)	34,022 (93)
	Hospital; number of samples (%)	2,568 (7)
Presence (+) or absence (−) of two mutations of interest	N501Y − and E484K −; number of samples	662
	N501Y + and E484K−; number of samples	31,929
	N501Y − and E484K +; number of samples	647
	N501Y + and E484K +; number of samples	3,352
Ct of the real-time reverse-transcription PCR (95%CI)		22.1 (14.9–29.4)
Sampling date (95%CI)		21 Apr (12 Apr–6 May)
Region	Normandie; number of samples	6,288
	Centre-Val de Loire; number of samples	1,961
	Hauts-de-France; number of samples	4,684
	Île-de-France; number of samples	16,922
	Occitanie; number of samples	409
	Provence-Alpes-Côte d'Azur; number of samples	3,789
	Bourgogne-Franche-Comté; number of samples	454
	Nouvelle-Aquitaine; number of samples	1,444
	Other; number of samples	639

CI: confidence interval.

## Sequencing profiles

The specificity of the variant-specific RT-PCR we used is limited, since this PCR only targets two mutations. To gain additional insights regarding the type of variants circulating in the country, we sequenced 15% the samples collected on 30 March 2021 in France in this dataset for which the Ct was equal or lower than 28 using Twist Libraries and Illumina sequencing; the GISAID accession numbers are in Supplement S1. These samples were constituted for the most part (45%; 215/478) by samples from the Île-de-France region and showed a majority of viruses of the B.1.1.7 lineage (79.1%, 378/478; Supplementary Table S1). The other prevalent lineages were B.1.351 (7.9%, 38/478), B.1.525 (4.4%, 21/478), and B.1.214 (2.3%, 11/478), a lineage characterised by a variant not classified as a VOC, but which is under monitoring [9]. There were also lineages represented by less than 2% of the samples, such as P.1 (0.6%, 3/478). In the subsets of samples from the Île-de-France (n = 215) and Hauts-de-France (n = 48) regions, the order of prevalence of the VOCs was the same as for the overall samples (Supplementary Table S1). Results from Santé Publique France, the French National Public Health Agency, for the Île-de-France region in April (n = 476 samples) also generally agreed with these findings (Supplementary Table S1). Only a few samples from April were sequenced from the Hauts-de-France (n = 11) and Île-de-France (n = 13) regions and for each of these regions, more than half of the samples were of the B.1.1.7 lineage.

Therefore, hereafter, samples with only the N501Y mutation detected are assumed to contain virus of B.1.1.7 lineage, samples with both N501Y and E484K mutations, mainly virus of the B.1.351 lineage with possibly a minority of P.1, samples with only the E484K mutation, virus of the B.1.525 lineage, and samples with no mutation, wild type SARS-CoV-2 (although these samples may contain also viruses of B.1.214 lineage, which also lack the two mutations).

## Analysis of reverse-transcription PCR results

**Figure 1:**
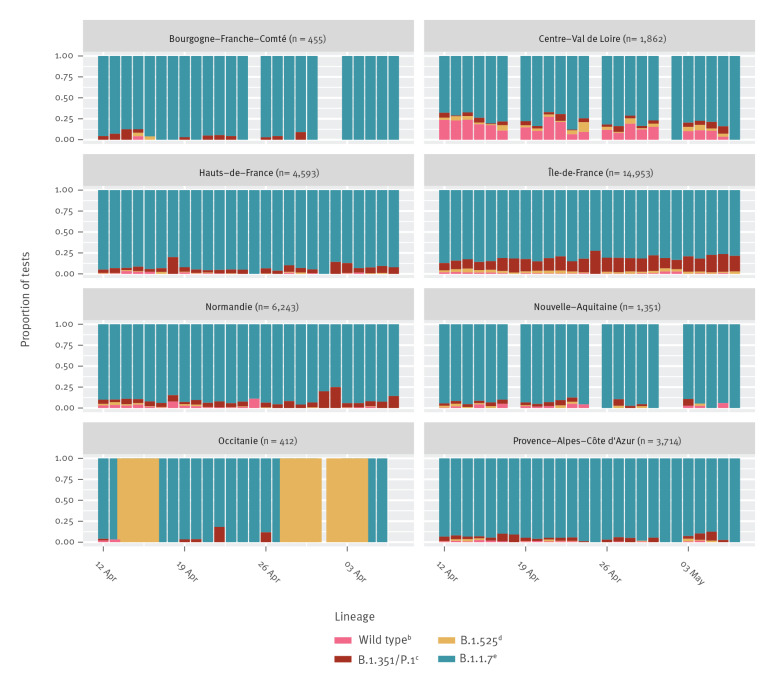
Raw daily cumulative frequencies of variant-specific reverse-transcription PCR test results for SARS-CoV-2 in eight French regions, 12 April–7 May 2021 (n = 33,583)^a^

SARS-CoV-2: severe acute respiratory syndrome coronavirus 2; VOC: variant of concern; VOI: variant of interest.

^a^ Only regions with more than 400 respective tests are shown.

^b^ Characterisation as wild type SARS-CoV-2 is based on the absence of both N501Y and E484K mutations.

^c^ Characterisation as B.1.351 and/or P.1 lineage (VOC β and/or γ) is based on the presence of both N501Y and E484K mutations.

^d^ Characterisation as B.1.525 lineage (VOI η) is based on the simultaneous absence of N501Y and presence of E484K mutation.

^e^ Characterisation as B.1.1.7 lineage (VOC α) is based on the simultaneous presence of N501Y and absence of E484K mutation.

Raw proportions of each SARS-CoV-2 lineage deduced by RT-PCR are shown in Figure 1 and raw numbers are shown in Supplementary Figure S1. As shown in both Figures, the sampling intensity in the dataset varies strongly across regions, which explains that some weeks have extreme values (e.g. in Occitanie). Overall, we see that lineage B.1.1.7 is dominant is most regions, and that the Île-de-France is the region where the B.1.351 and/or P.1 lineages are the most frequently detected.

The number of tests performed is indicated in each panel. For each day, the different colours indicate the proportion of tests belonging to each of the four screening categories (these sum to 1.0 every day). Regions with few tests can exhibit strong variations in frequencies for some days (e.g. Occitanie for days with only B.1.525 detected). See Supplementary Figure S1 for the raw numbers instead of the proportions.

## Ethical statement

This study has been approved by the Institutional Review Board of the CHU of Montpellier and is registered at ClinicalTrials.gov with identifier NCT04738331.

## Lineage spreading in France

We used a multinomial log-linear model (the multinom function from the nnet R package [10]) to identify factors associated with the detection of certain lineages (B.1.1.7 being the variant of reference). The explanatory variables were the individual’s age (type of variable: integer), origin of the sample (hospital or community; type of variable: binary), and the interaction between the sampling region (type of variable: categorical) and the calendar date of sampling (treated as an integer) (Table 2). Integer values were centred and scaled. Details about the statistical methods are presented in Supplement S2. The raw data and R script used are also provided as Supplementary data.

**Table 2:** Factors associated with the detection of certain SARS-CoV-2 lineages, as assessed by relative risk ratios using a multinomial log-linear model, France, 12 April–7 May 2021 (n = 36,590)

Factor		Median RRR with significance (95% CI) for the RRR		
		Wild type N501Y−/E484K−	B.1.351/P1 (VOC β/γ) N501Y+/E484K+	B.1.525 (VOI η) N501Y−/E484K+
Age (increase per year)		NS (0.88–1.00)	0.95^b^ (0.92–0.98)	0.86^b^ (0.80–0.93)
Origin of the samples	Non−hospital	Reference	Reference	Reference
	Hospital	NS (0.67–1.30)	1.56^a^ (1.40–1.80)	NS (0.64–1.30)
Interaction between sampling region and calendar date of sampling	Normandie	0.54^a^ (0.43–0.63)	NS (0.95–1.10)	0.69^a^ (0.55–0.81)
	Centre−Val de Loire	0.26^a^ (0.18–0.30)	NS (0.84–1.10)	NS (0.90–1.70)
	Hauts−de−France	0.72^b^ (0.57–0.91)	1.14^c^ (1.00–1.30)	NS (0.74–1.20)
	Île−de−France	0.74^a^ (0.64–0.82)	1.42^a^ (1.40–1.50)	0.81^a^ (0.71–0.91)
	Nouvelle−Aquitaine	NS (0.74–1.60)	1.25^c^ (1.10–1.50)	NS (0.57–1.20)
	Occitanie	NS (0.35–1.40)	NS (0.76–1.40)	NS (0.60–2.30)
	Provence−Alpes−Côte d'Azur	NS (0.67–1.10)	NS (0.95–1.20)	NS (0.74–1.20)
	Bourgogne-Franche-Comté	NS (0.38–1.70)	NS (0.55–1.10)	NS (0.34–1.50)
	Other	NS (0.50–1.70)	NS (0.80–1.40)	NS (0.42–1.40)

CI: confidence interval, NS: non-significant; VOC: variant of concern; VOI: variant of interest, RRR: relative risk ratios; SARS-CoV-2: severe acute respiratory syndrome coronavirus 2.

The minus (–) sign after a mutation indicates its absence; the plus (+) its presence.

The reference lineage in the analysis is B.1.1.7 (N501Y+/E484K−), i.e. the VOC α.

^a^ p < 0.001.

^b^ p < 0.01.

^c^ p < 0.05.

P values are obtained using a two-tailed z-test.

The multinomial model revealed differences between lineages (Table 2). In terms of age, we found that older patients had a lower risk of being infected by B.1.351/P.1 and B.1.525 than by B.1.1.7 (our reference). In hospital settings, we found an over-representation of B.1.351/P.1 compared with B.1.1.7. When analysing region-specific temporal trends, we found that, for all regions, the risks of being infected by a wild type or a B.1.525 virus were either identical or lower than the risk of being infected by B.1.1.7. Conversely, we found that the risk of being infected by B.1.351/P.1 instead of B.1.1.7 significantly increased with time in Île-de-France, and to a lesser extent in Hauts-de-France and Nouvelle-Aquitaine.

## Transmission advantage of B.1.351/P.1 vs B.1.1.7

Trends from the multinomial model should be treated with caution because of autocorrelation issues. Therefore, to investigate the temporal trends, we used the method described in [11] and, for each region of interest, fitted a logistic growth model to the fitted values of a generalised linear model (GLM) with three factors on the data sampled outside hospitals. In addition to the sampling date and the individual age, we also added the department (i.e. a within-region administrative unit), where the sample was performed. For simplicity, we tested the transmission advantage of B.1.351/P.1 compared with B.1.1.7 and neglected the other lineages in the analysis. We performed the analysis only in the Île-de-France, Hauts-de-France, and Nouvelle-Aquitaine regions. With few data regarding the coronavirus disease (COVID-19) epidemic serial interval in France, i.e. the time between the onset of the symptoms in an individual and that in a person he/she infects, we used the one from [12].

**Figure 2:**
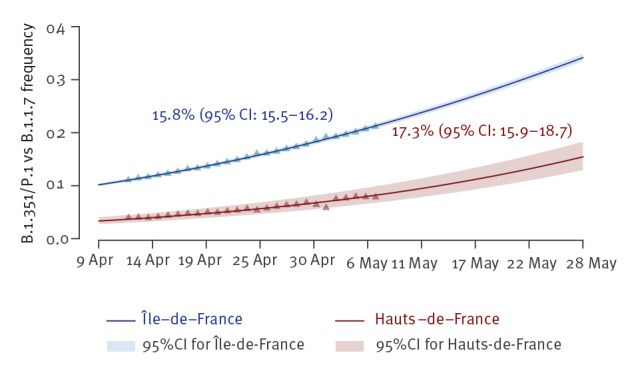
Estimated evolution of proportions of B.1.351/P.1^a^ (VOC β/γ) with respect to B.1.1.7 (VOC α), excluding other lineages, in Île-de-France and Hauts-de-France, France, 9 April–28 May 2021

CI: confidence interval; GLM: generalised linear model; VOC: variant of concern.

^a^ The reverse-transcription PCR used to detect the different variants cannot distinguish between P.1 (VOC γ) and B.1.351 (VOC β) lineages; based on the sequencing of a subset of samples in March and April however, the B.1.351 (VOC β) appears to dominate over the P.1 (VOC γ) lineage in France and in the regions considered. Therefore the results are considered as likely reflecting mostly the B.1.351 variant (VOC β) spread.

We found a transmission advantage of 15.8% (95% confidence interval: 15.5–16.2%) in Île-de-France and 17.3% (95% CI: 15.9–18.7%) in Hauts-de-France (Figure 2). In Nouvelle-Aquitaine, the logistic growth model was not significant, which could be due to the fact that this region was less affected by the third epidemic wave than the other two [13].

Each region is represented in a different colour, with the triangles indicating the GLM-fitted values and the line the output of the logistic growth model estimation. The shaded area in the same colour as the line represents the 95%CI. The text indicates the estimated transmission advantage of B.1.351 (VOC β) with respect to B.1.1.7 (VOC α) and the 95% CIs.

## Discussion

When analysing the results of variant-specific tests on samples obtained from January to March 2021, we found that the B.1.1.7’s (VOC α) transmission advantage relative to wild type lineages was larger than that of B.1.351 (VOC β) relative to wild type lineages [14]. During April 2021, in at least two French regions, this trend appears to have shifted with B.1.351 (VOC β) and possibly P.1 (VOC γ) spreading more rapidly than B.1.1.7. The B.1.351 lineage has known immune evasion properties [15,16]. Therefore, Île-de-France being one of the French regions the most impacted to date by the epidemic [13], it is possible that a shift in variants with a transmission advantage is occurring there, because of the high proportion of individuals with immunity acquired through prior-SARS-CoV-2-infections. Vaccination might favour immune escape mutants [17] but the coverage with COVID-19 vaccines is homogeneous among French regions. Our results call for more detailed analyses regarding the link between the transmission advantage of the B.1.351 variant and the proportion of the population with immunity (following infection or vaccination) in different French regions.

There are some limitations to this analysis. First, although we performed sequencing to distinguish between B.1.351 and P.1 lineages, in some samples collected in March and, to a lesser extent, April, further sequencing will be needed to validate our assumption that the transmission advantage belongs to B.1.351, to P.1, or to both. Second, France had entered a third national lockdown on 3 April, which means that most of the tests analysed here were performed in a declining epidemic [18]. If what we assume to be the B.1.351 lineage causes infections that have a shorter generation interval than the B.1.1.7 lineage, this could affect the transmission advantage estimates. While there are some data on generation intervals for COVID-19 epidemics in France [19], studies are so far limited. Furthermore, analyses performed on the detailed United Kingdom epidemic data found that the hypothesis of differences in generation interval between B.1.1.7 and wild type lineages was less likely than other hypotheses, especially differences in contagiousness [2]. Finally, it is unlikely that non-pharmaceutical interventions would affect differently the transmission of the variants.

In conclusion, given the progressive lifting of the control measures in June 2021 in France [18], these results call for particular care regarding vaccination rollout and the maintenance of non-pharmaceutical prevention until vaccine coverage reaches levels compatible with spontaneous regression of the epidemic.

## Supplementary Data

12000 Supplement_S1

## Supplementary Data

229000 Supplement_S2

## Supplementary Data

45000 Supplementary_FigureS1

## Supplementary Data

21000 Supplementary_TableS1

## Supplementary Data

195000 Supplementary_data
