# Supplementary material for: The SARS-CoV-2 B.1.351 lineage (VOC β) is outgrowing the B.1.1.7 lineage (VOC α) in some French regions in April 2021
Source: Euro Surveill. 2021 Jun 10;26(23):2100447. doi: 10.2807/1560-7917.ES.2021.26.23.2100447 (PMC8193991; doi:10.2807/1560-7917.ES.2021.26.23.2100447)
Supplement: Supplement_S2 [file 2100447_Supplement_S2.pdf]

This supplementary material is hosted by Eurosurveillance as supporting information alongside the article « The SARS-CoV-2 B.1.351 lineage (variant β) is outgrowing the B.1.1.7 lineage (variant α) in French regions in April 2021 *» on behalf of the authors who remain responsible for the accuracy and appropriateness of the content. The same standards for ethics, copyright, attributions and permissions as for the article apply. Supplements are not edited by Eurosurveillance and the journal is not responsible for the maintenance of any links or email addresses provided therein.*

Supplementary Methods

Multinomial log-linear model

To perform the multinomial log-linear model, we used the multinom function from the nnet R package. This function uses neural networks to perform model selection in a step wise manner starting from the null model (i.e. without any predictor).

The model formula was the following: `variant ~ age + location_sampling + date:region`, where `age` is the age of the individual (which is treated as an integer and centered and scaled), `location_sampling` is a binary variable indicating whether the sample was collected in a hospital or not, `date` is the sampling date (which is treated as an integer and centered and scaled), and `region` is the French administrative region of sampling.

The multinom function uses an AIC criterion to identify the best model and returns the estimated multinomial logistic regression coefficients as well as their standard error (SE).

These can be used to calculate a z test statistic, which is simply the ratio of the coefficient value to the SE. From there, we can construct a p-value, P>|z|, which is the probability the z test statistic would be observed under the null hypothesis and assuming that z follows a normal distribution. Here, we use a classical significance threshold of alpha 5%. When the p-value is smaller than alpha, the null hypothesis can be rejected and the parameter is considered to be significant.

Note that an alternative approach could be to calculate the 95% confidence interval for the coefficient value of the multinomial model using the SE and a critical value on the standard normal distribution.

To give a more intuitive interpretation of the results, we compute the relative risk ratios (RRR) by taking the exponential of the coefficient values of the model. The RRR reflects, for a given variable, how the risk of belonging to one of the outcomes (here variant detection) varies compared to the control group.

Further details about multinomial log-linear models and their interpretations can be found at [1].

Selection advantage estimation

Following methods developed in population genetics to estimate the selection coefficient of a mutant allele compared to a wild type allele [2], and following earlier studies in epidemiology [3-5], we calculate the selection coefficient s by fitting a logistic growth model to the time series of variant frequency.

Indeed, provided that the selection coefficient s does not vary over time, and by denoting p(t) the frequency of an allele (here variants V2 or V3) in the population (here variants V1, V2, and V3), we have the following relationship:

$$s = \frac{d}{dt} log\left(\frac{p(t)}{1 - p(t)}\right)$$

Note that this value needs to be scaled with respect to the generation time T, which is here obtained from the serial interval calculated by [6]. Overall, the transmission advantage s_T of variants V2 and V3 over variant V1 is given by the formula $s_T = s\ T$.

In order to estimate s, for each region of interest separately, we first perform a generalised linear model (GLM) with a binomial distribution of the residuals (i.e. a logistic regression) where the response variable is the variant type (V2/V3 or V1) and the factors are the `age` of the individual (which is treated as an integer and centered and scaled), the sampling `date` (which is treated as an integer and centered and scaled), and the sampling `department`, which is the French administrative level below the region. We then use the fitted values from the GLM to perform the fit of the logistic growth function.

Further details about the implementation of the inference can be found in the Supplementary R script with the Supplementary data.

References

[1] Multinomial logistic regression | STATA annotated output, https://stats.idre.ucla.edu/stata/output/multinomial-logistic-regression/ (accessed June 4, 2021)

[2] Chevin L-M. 2011. On measuring selection in experimental evolution. *Biology Letters* 7:210–213. DOI: 10.1098/rsbl.2010.0580.

[3] Davies NG, Abbott S, Barnard RC, Jarvis CI, Kucharski AJ, Munday JD, Pearson CAB, Russell TW, Tully DC, Washburne AD, Wenseleers T, Gimma A, Waites W, Wong KLM, Zandvoort K van, Silverman JD, Group1‡ CC-19 W, Consortium‡ C-19 GU (COG-U, Diaz-Ordaz K, Keogh R, Eggo RM, Funk S, Jit M, Atkins KE, Edmunds WJ. 2021. Estimated transmissibility and impact of SARS-CoV-2 lineage B.1.1.7 in England. *Science*:eabg3055. DOI: 10.1126/science.abg3055.

[4] Volz E, Mishra S, Chand M, Barrett JC, Johnson R, Geidelberg L, Hinsley WR, Laydon DJ, Dabrera G, O'Toole Á, Amato R, Ragonnet-Cronin M, Harrison I, Jackson B, Ariani CV, Boyd O, Loman NJ, McCrone JT, Gonçalves S, Jorgensen D, Myers R, Hill V, Jackson DK, Gaythorpe K, Groves N, Sillitoe J, Kwiatkowski DP, Flaxman S, Ratmann O, Bhatt S, Hopkins S, Gandy A, Rambaut A, Ferguson NM. 2021. Assessing transmissibility of SARS-CoV-2 lineage B.1.1.7 in England. *Nature*:1–17. DOI: 10.1038/s41586-021-03470-x.

[5] Haim-Boukobza S, Roquebert B, Trombert-Paolantoni S, Lecorche E, Verdurme L, Foulongne V, Selinger C, Michalakis Y, Sofonea MT, Alizon S. 2021. Detection of Rapid SARS-CoV-2 Variant Spread, France, January 26--February 16, 2021. *Emerging Infectious Diseases* 27:1496–1499. DOI: 10.3201/eid2705.210397.

[6] Nishiura H, Linton NM, Akhmetzhanov AR. 2020. Serial interval of novel coronavirus (COVID-19) infections. *International Journal of Infectious Diseases* 93:284–286. DOI: 10.1016/j.ijid.2020.02.060.
