# Supplementary material for: The SARS-CoV-2 B.1.351 lineage (VOC β) is outgrowing the B.1.1.7 lineage (VOC α) in some French regions in April 2021
Source: Euro Surveill. 2021 Jun 10;26(23):2100447. doi: 10.2807/1560-7917.ES.2021.26.23.2100447 (PMC8193991; doi:10.2807/1560-7917.ES.2021.26.23.2100447)

This supplementary material is hosted by Eurosurveillance as supporting information alongside the article
« The SARS-CoV-2 B.1.351 lineage (variant β) is outgrowing the B.1.1.7 lineage (variant α) in French regions in April 2021 »
on behalf of the authors who remain responsible for the accuracy and appropriateness of the content.
The same standards for ethics, copyright, attributions and permissions as for the article apply.
Supplements are not edited by Eurosurveillance and the journal is not responsible for the maintenance
of any links or email addresses provided therein.

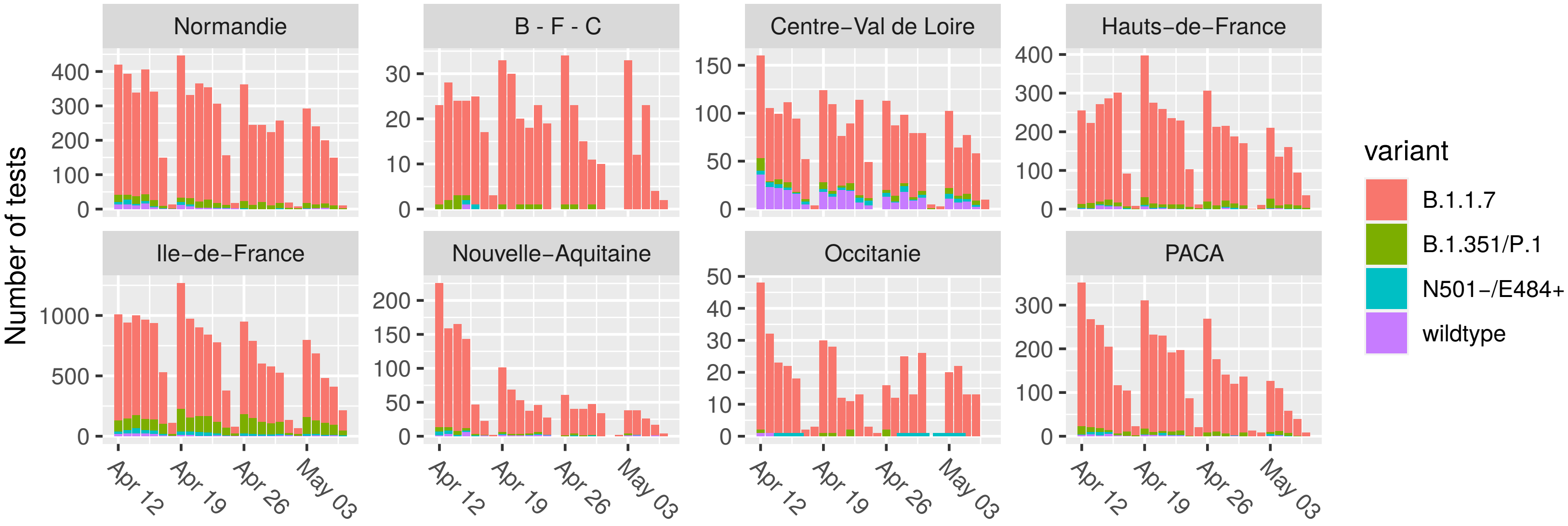

Supplement: Supplementary_FigureS1 [file 2100447_Supplementary_FigureS1.pdf]
